# An antibody-dependent cellular phagocytosis-related gene signature predicts survival and response to immunotherapy in stomach adenocarcinoma

**DOI:** 10.1097/MD.0000000000042079

**Published:** 2025-04-04

**Authors:** Xiaochuan Li, Hongjian Wang, Xiaofeng Li, Miaoen Zeng, Zhuguang He, Linjie Song, Zhiming Chen, Xinyue Tang, Ang Wang

**Affiliations:** a Department of Colorectal and Anorectal Surgery, Qingdao Hospital, University of Health and Rehabilitation Sciences (Qingdao Municipal Hospital), Qingdao, China; b General Surgery Department of Yunfu People’s Hospital, Yunfu, China; c Guangdong Second Provincial General Hospital, Guangzhou, China; d Department of Gastroenterology, Fogang County People’s Hospital, Qingyuan, China; e Department of Oncology, Zhaoqing First People’s Hospital, Zhaoqing, China; f Department of Integrated Traditional Chinese and Western Medicine, Cancer Hospital of Shantou University Medical College, Shantou, China; g Department of Oncology, Guangdong Second Provincial General Hospital, Guangzhou, China.

**Keywords:** ADCP, gene signature, immunotherapy, STAD, survival

## Abstract

Antibody-dependent cellular phagocytosis (ADCP) is an immune biological process and plays a biological role in the clearance of tumor cells and the response to immune checkpoint inhibitors. However, the effects of ADCP on stomach adenocarcinoma (STAD) remain unclear. Clinical and genomic data were extracted from multiple datasets. The ADCP-related signature was established using Cox least absolute shrinkage and selection operator regression. Expression of the C5a receptor also known as complement component 5a receptor 1 in the tumor and adjacent-normal tissues was calculated using immunohistochemistry staining. Validation of the signature was conducted in the training and validation cohorts by Cox regression and log-rank tests. Furthermore, the immune infiltrates, the tumor immune dysfunction and exclusion score, and tumor mutation burden score were calculated using the corresponding algorithms, and Mann–Whitney *U* tests were used to evaluate the differences between groups. Seventy-three hub genes with predictive performance were identified to establish an ADCP-related signature. Accordingly, a 27-gene signature was established, C5a receptor also known as complement component 5a receptor 1, one of the signature genes, had higher expression in tumors than adjacent-normal samples, and its predictive performance was validated in the GSE84437 and The Cancer Genome Atlas cohorts. We found that the ADCP-related signature is an excellent prognostic predictor of STAD. Moreover, the molecular characteristics and some indices of response to immunotherapy differed between the high- and low-risk groups. We constructed a 27-gene signature that is associated with the prognosis and response to STAD-based immunotherapy and provide insights into the biological mechanisms underlying this predictive function.

## 1. Introduction

Stomach adenocarcinoma (STAD) accounts for approximately 95% of gastric cancer cases in clinical practice and ranks third among cancer-related deaths worldwide.^[1]^ Despite recent advancements in diagnostic techniques and therapeutic medication, the survival of patients with STAD remains unsatisfactory.^[2]^ Therefore, it is crucial to identify novel prognostic biomarkers to guide clinical diagnosis and treatment. Recently, immunotherapy has become an option for patients with locally advanced or metastatic STAD.^[3,4]^ Immune checkpoint-blocking agents, such as immune checkpoint inhibitors (ICIs), have shown remarkable therapeutic effects in STAD. However, not all patients with STAD respond positively to ICIs.^[5]^ The identification of new biomarkers, in addition to microsatellite instability and tumor mutation burden (TMB), is crucial for improving ICI outcomes in patients with STAD.

Antibody-dependent cellular phagocytosis (ADCP) is an immune biological process in which specific antibodies bind to both target cells, such as tumor cells, and effector cells, such as macrophages, thereby inducing phagocytosis of the target cells by the effector cells.^[6]^ Once phagocytosis occurs, the target cells are digested and degraded within effector cells due to acidification.^[7]^ In addition to enabling the immune system to eliminate cancer cells, ADCP also functions as a crucial mediator of ICIs.^[8]^ The Fc (fragment, crystallizable) segment of some ICIs can bind to FcγR in macrophages, enabling them to identify and eliminate tumor cells, thereby fulfilling its role in tumor clearance.^[7]^ However, the effect of ADCP on the prognosis of gastric cancer and its utility in identifying patients with STAD who may benefit from ICIs in STAD remain unknown. This study aimed to assess the performance of ADCP in predicting the prognosis of STAD and the efficacy of ICI treatment in STAD, using a bioinformatics approach.

## 2. Methods

### 2.1. Data acquirement and processing

Genomic data (RNA-seq data and mutation data) and corresponding clinical information (including age, sex, TNM stage, tumor stage, and survival time) of STAD were downloaded from UCSC-Xena platform (https://toil.xenahubs.net).^[9]^ The expression profiles of 431 STAD samples with the clinical information of the 483 samples in GSE84437 dataset was downloaded from the Gene Expression Omnibus (http://www.ncbi.nlm.nih.gov/geo/). Annotated and standardized probe expression matrices from the databases were used in this study. A list of 511 ADCP genes was obtained from a previously published article.^[10]^ We also obtained a list of immune checkpoint-related genes (such as PDCD1, CD274, CTLA4, HAVCR2, BTLA, TIGIT, SIRPA, TNFRSF4, TNFRSF9, ICOS, LAG3, CD47, and VTCN1) and HLA family genes from previously published literature.

### 2.2. Selection of survival-related ADCP genes and functional analysis

The GSE84437 dataset was used as the training cohort in the present study. Univariate Cox regression analysis on the ADCP genes was performed using the “survival” and “survminer” packages in R, and genes with a *P*-value of < .05 were selected as a predictor variable. Moreover, the clusterProfiler R package was used to perform gene ontology, including biological process, cellular component, molecular function, and Kyoto Encyclopedia of Genes and Genomes pathway enrichment analysis based on the survival-related ADCP genes. *P*-vlaue < .05 indicated the significant enrichment.

### 2.3. Establishment and validation of ADCP-related prognostic signature

Least absolute shrinkage and selection operator (LASSO) Cox regression with 5-fold cross-validation was performed to the establishment of prognostic signature in the significant ADCP genes associated with survival prognosis obtained in the previous step using the “glmnet” package. Based on the results of LASSO Cox regression, the prognostic signature was established as follows: risk score = ∑β_gene_ × Exp_gene_. With β_gene_ representing the coefficient of the gene and Exp_gene_ representing the gene expression level in the GSE84437 dataset. Then, time-dependent receiver operating characteristic curves were plotted to evaluate the predictive performance of the ADCP-related signature in GSE84437 cohorts using the “timeROC” package. The cutoff value was identified using the “surv_cutpoint” function and the patients in the GSE84437 cohort were divided into 2 groups. The overall survival (OS) between the 2 groups was plotted using the Kaplan–Meier curve method with the “survminer” package and compared using the log-rank test. The ADCP-related risk score by 27-gene signature was subjected to univariate and multivariate Cox regression analyses using the “survival” R package, and the nomogram and its calibration curve were plotted using the “rms” package. The Cancer Genome Atlas (TCGA)-STAD cohort was used as the validation set.

### 2.4. Gastric carcinoma tissue microarray and immunohistochemistry (IHC)

The gastric carcinoma tissue microarray was obtained from Shanghai Outdo Biotech Company, Ltd. The microarray was immune-stained by IHC methods to detect the expression of C5a receptor also known as complement component 5a receptor 1 (C5AR1). Briefly, the microarray was deparaffinized with dimethylbenzene and ultrapure water. After retrieving the antigens through heating treatment for 10 minutes and air cooling for 30 minutes. Then, endogenous peroxidase was inactivated with 3% hydrogen peroxide for 10 minutes, followed by blocking with 10% FBS for 1 hour. Microarray was incubated with a primary C5AR1 antibody (1:100; code: 124365, Chengdu, China) and a second antibody at room temperature for 2 to 3 hours. The H-Score was calculated based on a formula as follows, H-SCORE = ∑(pi × i) = (percentage of weak intensity × 1) + (percentage of moderate intensity × 2) + (percentage of strong intensity × 3). GraphpadPrism software was used for data processing and visualization.

### 2.5. Identification of differentially expressed genes (DEGs), construction of protein–protein interaction (PPI) network

The “limma” package was used to distinguish DEGs between different risk groups. Genes with adjusted *P* < .05 and |log2Fold change (FC)| > 0.5 were considered DEGs. An online tool, STRING (Version:11.0, http://string-db.org/) was used to construct a PPI network with a connection score of more than 0.7, and the network was constructed using Cytoscape 3.6.1 (https://cytoscape.org/) applications.

### 2.6. Gene set enrichment analysis (GSEA)

For GSEA, GSEA software (version 3.0) was downloaded from the GSEA website (http://software.broadinstitute.org/gsea/index.jsp). The c2.cp.kegg.v7.4. symbols.gmt subset was then downloaded from the Molecular Signatures Database (http://www.gsea-msigdb.org/gsea/downloads.jsp) to identify enriched pathways in the GSEA analysis. The analysis was performed with a minimum gene set size of 5 and a maximum gene set size of 5000, and with 1000 resampling. A threshold of *P* < .05 was established as the criterion for determining statistical significance.

### 2.7. Development of a predictive nomogram

Univariate regression analysis was performed to evaluate the prognostic value of gene signatures and clinicopathological features, Multivariate Cox regression analysis subsequently was performed on independent prognostic factors. Afterward, the rms package was used to develop a nomogram for predicting OS. The calibration plot was described to assess the accuracy and consistency of the predictive model.

### 2.8. Correlations between somatic mutation, immune cell infiltration, immune checkpoint-related gene expression, HLA genes, and ADCP-related risk score

The “maftools” package was used to create the waterfall plot of the genetic variations. The TMB of the tumor tissue was also calculated using data downloaded from the UCSC-Xena platform. Correlation analysis was conducted with the “Hmisc” package between the TMB and ADCP-related risk scores. The composition of 22 immune cells using CIBERSORT was compared between the 2 groups using the Mann–Whitney *U* test (https://cibersort.stanford.edu). Furthermore, the stromal score and immune score estimated using the “ESTIMATE” package were also compared between the 2 groups using the Mann–Whitney *U* test. Moreover, the differentially expressed the immune checkpoint-related genes (such as PDCD1, CD274, CTLA4, ICOS, HAVCR2, LAG3, CD47, BTLA, TIGIT, SIRPA TNFRSF4, TNFRSF9, and VTCN1) and HLAs between 2 groups were determined using the Mann–Whitney *U* test.

### 2.9. Correlation between the chemotherapy and immunotherapy and ADCP-related risk

Based on the Genomics of Drug Sensitivity in Cancer (https://www.cancerrxgene.org/), the half maximal inhibitory concentration (IC50) value was calculated using the pRRophetic package. The differences in IC50 values between the 2 groups were determined using the Mann–Whitney *U* test. The higher IC50 value indicated the resistance to chemotherapy. Moreover, the tumor immune dysfunction and exclusion (TIDE, http://tide.dfci.harvard.edu/) was evaluated, and the differences in TIDE score between the 2 groups were determined using the Mann–Whitney *U* test. A higher TIDE score indicated the immunosuppressive state.

### 2.10. Statistical analysis

Raw RNA-seq data were standardized using Log_2_ (count + 1). The Mann–Whitney *U* test or Kruskal–Wallis test with post hoc Dunn test was used to evaluate continuous variable data. All statistical analyses were conducted using R software (version 4.1) or GraphpadPrism software. Statistical significance was set at *P* < .05.

## 3. Results

### 3.1. Identification of the survival-related ADCP genes

**Figure 1.**
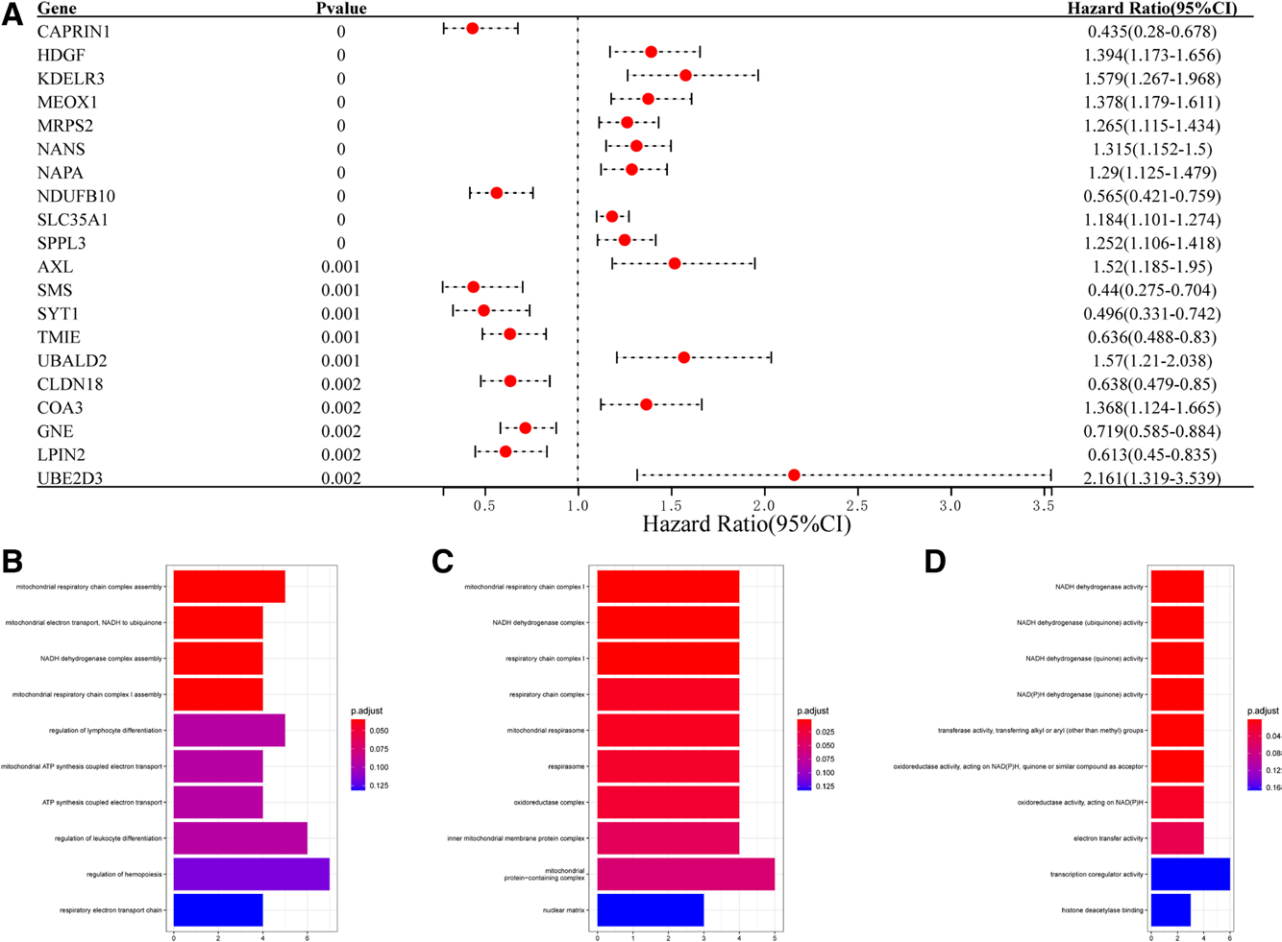
Identification of the survival-related ADCP genes. (A) 73 genes with significant predictive performance in GSE84437 were identified by univariate COX regression (the picture shows the results of the top 20 ranked by *P*-value). (B)–(D) GO analysis showed that a total of 328 GO BP, 27 GO CC, and 36 GO MF were enriched (the picture shows the results of the top 10 ranked by *P*-value). ADCP = antibody-dependent cellular phagocytosis, BP = biological process, CC = cellular component, GO = gene ontology, MF = molecular function.

A total of 431 samples from GSE84437 cohort with survival information were analyzed in this study. After merging the mRNA profiles from GSE84437 cohort and 511 ADCP-related genes, 478 ADCP-related genes were incorporated for subsequent analyses. In the GSE84437 cohort, a univariate Cox regression analysis was conducted on 478 ADCP-related genes. A total of 73 genes with significant predictive performance were identified (Table S1, Supplemental Digital Content, http://links.lww.com/MD/O621, Fig. 1A, which shows the top 20 genes ranked by *P*-value). GO analysis was conducted on these 73 genes, and 328 terms in biological process, 27 terms in cellular component, and 36 terms in molecular function were enriched (Fig. 1B–D, showing the top 10 ranked results based on the *P*-value).

### 3.2. Construction and validation of the ADCP-related signature in STAD

**Figure 2.**
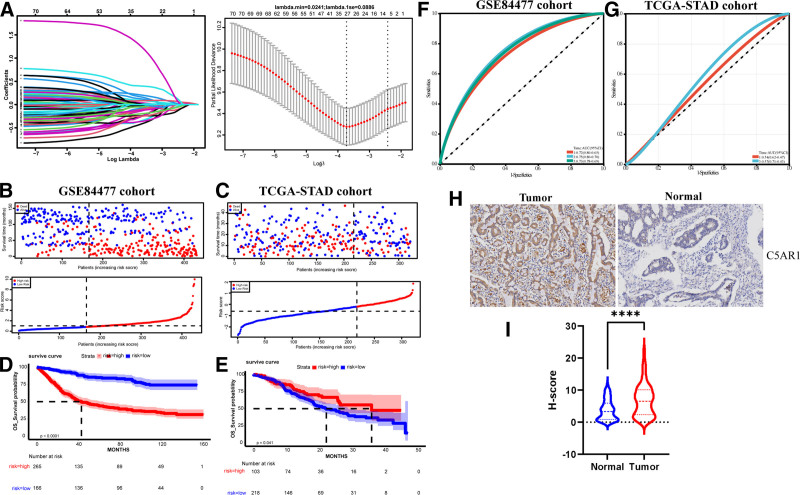
Construction and validation of the ADCP-related signature in STAD. (A) By LASSO COX regression with 5-fold cross-validation, 6 genes correlated with prognosis were identified. (B) and (C) The score with the highest sensitivity and specificity at 3-year OS was selected as the cutoff in GSE84437 and TCGA-STAD cohorts. (D) and (E) Survival time between high-risk group and low-risk group in GSE84437 and TCGA-STAD cohorts. (F) and (G) Time-dependent ROC at 1, 3, and 5 years of risk score for OS in GSE84437 and TCGA-STAD cohorts. (H) and (I) IHC analysis of the C5AR1 expression in STAD tumor and adjacent-normal tissues. IHC = immunohistochemistry, LASSO = least absolute shrinkage and selection operator, OS = overall survival, STAD = stomach adenocarcinoma, TCGA = The Cancer Genome Atlas.

LASSO Cox regression analysis was performed to select the ADCP-related signature, including PDSS2, STAG2, BCOR, SEL1L3, HMBS, SYT1, NDUFB10, TMIE, C5AR1, LAG3, RAB1B, PRKCD, GK5, NDUFS7, AK2, KRT23, COA3, SEC23B, WDR1, NDUFA1, SLC35A1, INTS5, CLCC1, IKZF3, SCN1B, UBE2D3, VPS72 (Fig. 2A). The ADCP-related risk score was calculated based on the partial regression coefficients and the RNA expression of the 27 signature genes. All patients were divided into high-risk and low-risk groups in the training set (GSE84437) and validation set (TCGA-STAD) with the median risk score (Fig. 2B and C). The log-rank test showed that the OS was significantly different between the 2 groups (Fig. 2). In the GSE84437 cohort, time-dependent ROC was plotted, and the area under the curve values of the ADCP-related risk score at the 1st, 3rd, and 5th year were 0.72, 0.75, and 0.73 (Fig. 2F). Finally, the robustness of the results was validated in the TCGA-STAD cohort (Fig. 2E and G). Importantly, C5AR1 is one of the signature genes in STAD in the present study, which plays an important role in the tumorigenesis in several tumors.^[11–13]^ Therefore, we focused on the expression of the C5AR1 in STAD. The IHC assay results also demonstrated that C5AR1 expression was significantly upregulated in the tumor samples compared with the adjacent-normal samples (Fig. 2H and I), indicating that expression of C5AR1 was strongly increased in the STAD and associated with tumorigenesis.

### 3.3. DEGs screening and biological function analysis in high-risk and low-risk groups

**Figure 3.**
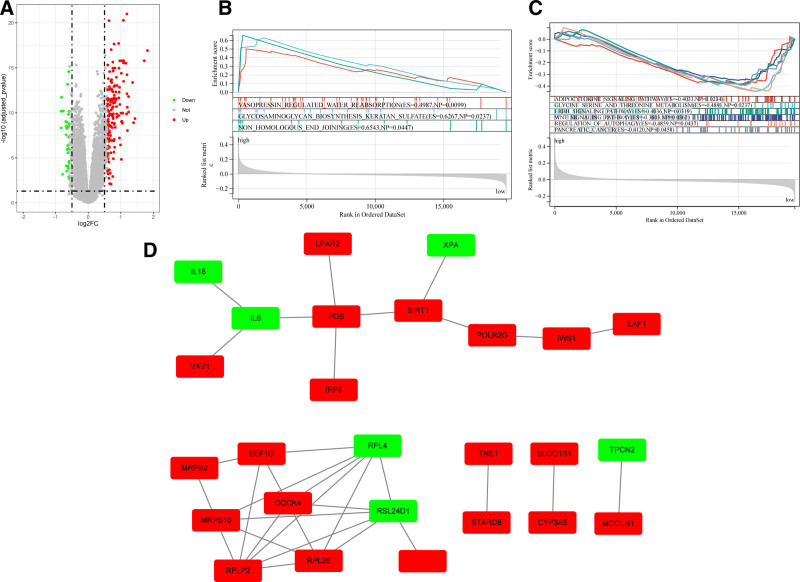
DEG screening and biological function analysis in high-risk and low-risk groups. (A) DEGs between high-risk and low-risk groups in STAD. (B) and (C) The result of GSEA analysis. (D) and (E) Dividing patients into 3 categories was most appropriate. (D) PPI network based on DEGs in high-risk and low-risk groups. DEGs = differentially expressed genes, GSEA = gene set enrichment analysis, PPI = protein–protein interaction, STAD = stomach adenocarcinoma.

A total of 192 DEGs (162 upregulated and 30 downregulated, Table S2, Supplemental Digital Content, http://links.lww.com/MD/O622) in the 2 groups were identified (Fig. 3A). And GSEA was performed, we found that the significantly upregulated genes were enriched in vasopressin-regulated water reabsorption, glycosaminoglycan biosynthesis, keratan sulfate, and non-homologous end-joining pathways, and the significantly downregulated genes were enriched in adipocytokine signaling, glycine serine and threonine metabolism, ERBB signaling, Wnt signaling, and regulation of autophagy pathways (Fig. 3B and C). Moreover, a PPI network was constructed based on DEGs, 2 sub-networks were identified (Fig. 3D), and 1 sub-network contained 8 upregulated DEGs (VAV1, LPAR2, FOS, IRF9, SIRT1, POLR2G, IWS1, and EAF1) and 3 downregulated DEGs (IL6, IL8, and XPA). Another sub-network contained 7 upregulated DEGs (MRPS2, EEF1G, MRPS10, DOCK4, RPLP2, RPL26, and GNL3L) and 2 downregulated DEGs (RPL4 and RSL24D1).

### 3.4. Development of a predictive nomogram

**Figure 4.**
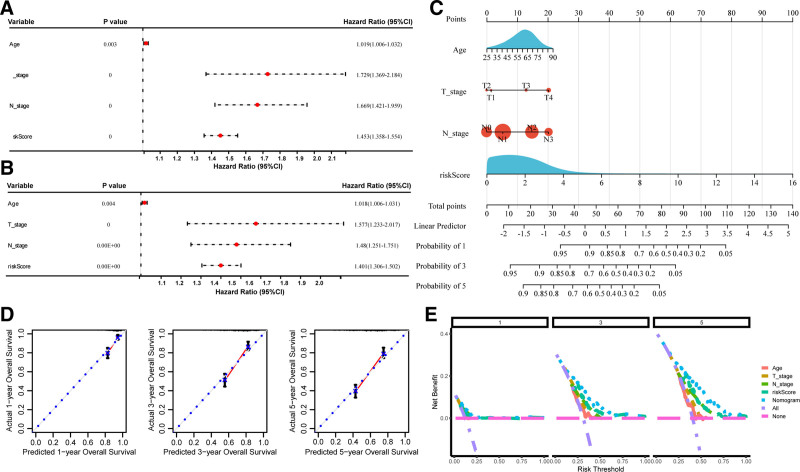
Development of a predictive nomogram. (A) Univariate Cox regression analysis showed that the patients with higher risk scores experienced a higher hazard rate of death (HR = 1.453, 95% CI: 1.358–1.554). (B) Multivariate Cox regression analysis found that the patients with higher risk scores showed a higher hazard rate of death (HR = 1.401, 95% CI: 1.306–1.502). (C) The nomograph is based on the results of the multivariate Cox regression analysis. (D) The calibration curve. (E) The decision curves. HR = hazard ratio, OS = overall survival.

We then explored the association between ADCP-related risk scores and OS. Univariate analysis using Cox regression (Fig. 4A) revealed that patients with higher scores had a higher hazard ratio (HR) of death (HR = 1.453, 95% CI: 1.358–1.554). Multivariate Cox regression analysis was further conducted (Fig. 4B), revealing that patients with higher scores showed a higher HR of death (HR = 1.401, 95% CI: 1.306–1.502). These results indicate that the ADCP-related risk score is an independent predictor of OS in STAD. The nomogram was plotted according to the results of the multivariate Cox regression analysis, and the observed and predicted 3-year OS showed good consistency in the calibration curve (Fig. 4C and D). And the decision curves indicated that the nomogram provided optimal clinical net benefit for 1-, 3-, 5-year OS (Fig. 4E).

### 3.5. Mutant landscape in high-risk and low-risk groups in the TCGA cohort

**Figure 5.**
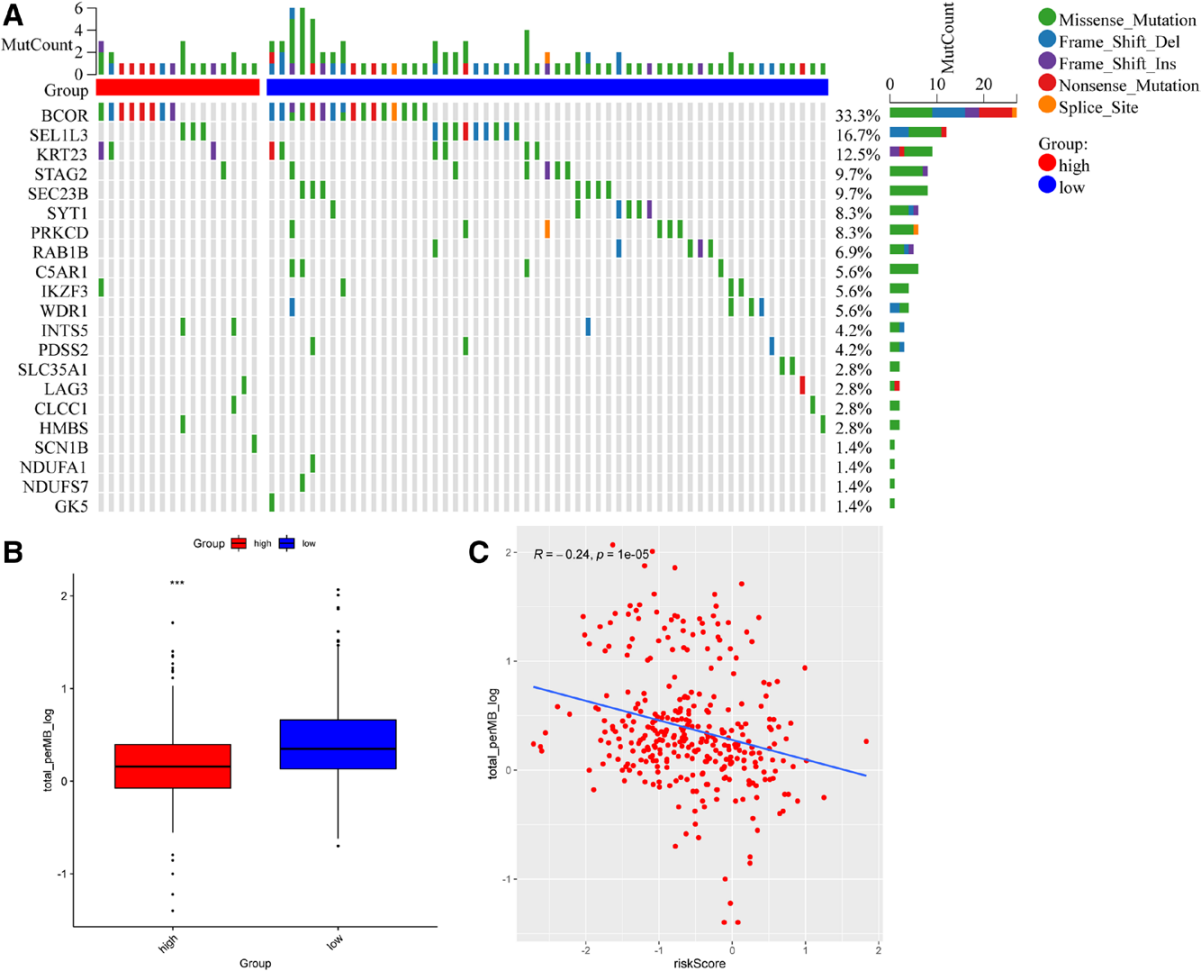
Mutant landscape in high-risk and low-risk groups in the TCGA cohort. (A) The waterfall plot of the mutated genes in both the high- and low-risk groups. (B) The boxplot of the TMB between high-risk and low-risk groups. (C) Scatter plots of the correlation of risk score and TMB. TCGA = The Cancer Genome Atlas, TMB = tumor mutation burden.

TMB and mutation are significantly associated with response to immunotherapy, high TMB reveals the high sensitivity to immune checkpoint inhibitors.^[14,15]^ Here, we found the number of mutated genes in STAD (Fig. 5A), such as BCOR (33.3%), SEL1L3 (16.7%), and KRT23 (12.5%). We also observed a lower TMB in the high-risk group compared to the low-risk group (Fig. 5B). Correlation analysis demonstrated the negative correlation between TMB and risk score in STAD (Fig. 5C). These results indicate that the high-risk group with high TMB might be associated with resistance to immunotherapy.

### 3.6. Landscape immune cell infiltration landscape in high-risk and low-risk groups

**Figure 6.**
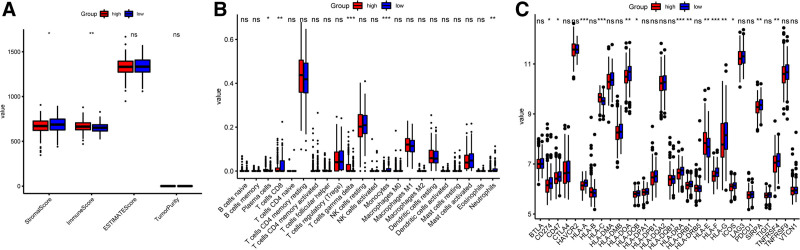
Landscape immune cell infiltration landscape in high-risk and low-risk groups. (A) Tumor purity, ESTIMATE score, Stroma score, and immune score between high-risk and low-risk groups. (B) COBESORT algorithm indicates the 22 types of immune cell infiltrating between high-risk and low-risk groups. *P < .05, **P < .01, ***P < .001, ****P < .0001, and ns means *P* > .05. (C) The differences of ICRGs and HLAs between high-risk and low-risk groups. ICRG = immune checkpoint-related gene.

To explore the different characteristics of the tumor immune microenvironment between the different risk groups, ESTIMATE algorithm results indicated that decreased Stroma score and increased Immune score in a high-risk group than the low-risk group (Fig. 6A). Then, the CIBERSORT algorithm was used to calculate the infiltration levels of immune cells in the tumors. The infiltration levels of 22 immune cell types were analyzed, and a Mann–Whitney *U* test was conducted. Several immune cells showed significantly different levels of infiltration between the 2 groups (Fig. 6B). The numbers of CD8 + T cells, monocytes, and neutrophils in the tumors were significantly different between the 2 groups, indicating that the different prognoses in these groups may be linked to the levels of these immune cells in the tumor. We explored the predictive performance of the ADCP-related signature for the response to immunotherapy and compared the RNA expression of genes, including those in the HLA family and those associated with the response to ICIs in the 2 groups. The results revealed a significant low in the RNA expression levels of CD274, CD47, HLA-A, HLA-DOA, HLA-DOB, HLD-DRA, HLA-DRB1, HLA-F, HLA-G, ICOS, SIRPA, and TNFRSF4 in high-risk than low-risk group (Fig. 6C).

### 3.7. Correlation of therapeutic response and risk score

**Figure 7.**
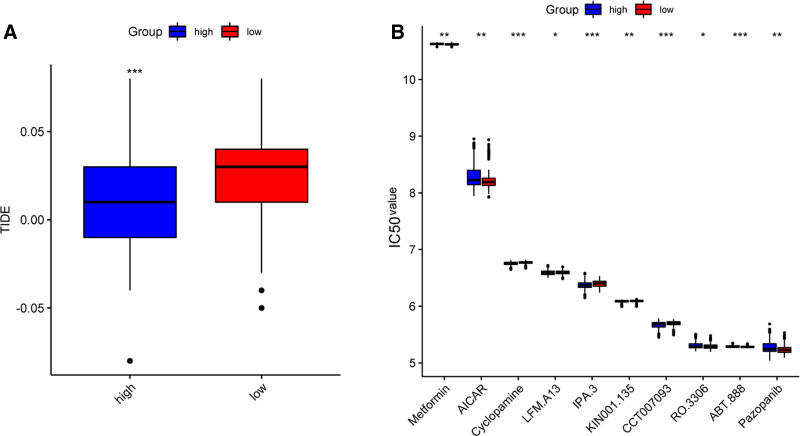
Correlation of therapeutic response and risk score. (A) The differences in TIDE score between high-risk and low-risk groups. (B) The differences in IC50 values between high-risk and low-risk groups based on the GDSC database. GDSC = The Genomics of Drug Sensitivity in Cancer, IC50 = the half maximal inhibitory concentration, TIDE = the tumor immune dysfunction and exclusion.

The TIDE score, an index used to predict the response of patients to immunotherapy, was also significantly higher in the low-risk group (Fig. 7A). Although there was no validation cohort, these results indicate that patients with lower ADCP-related scores might experience a better response to immunotherapy than those with higher scores. The response to chemotherapy analysis indicated that higher IC50 values of Metformin, AICAR, RO.3306, ABT.888, and Pazopanib, whare lower IV50 values of Cyclopamine, LFM.A13, IPA.3, KIN001.135, and CCT007093 in low-risk group than high-risk group (Fig. 7B).

## 4. Discussion

ADCP is an immune response process mediated by immune cells that promotes the phagocytosis of cells labeled with specific antibodies by effector cells.^[6]^ When cancer cell antigens are detected, the immune system is activated, leading to the activation and differentiation of B cells that produce specific antibodies.^[7]^ These antibodies bind to antigens on the surface of cancer cells and mark their destruction by immune cells, such as macrophages, which engage with cancer cells via antibodies and activate ADCP.^[7]^ Li et al demonstrated that a stronger ADCP effect was positively correlated with more favorable survival in patients with clear cell renal cell carcinoma.^[10]^ Furthermore, van der Horst et al reported that enhancing the ADCP effect using a monoclonal antibody presents a promising treatment for B-cell lymphomas.^[16]^ ADCP is a key effector mechanism in neuroblastoma therapy, particularly when using anti-disialoganglioside monoclonal antibodies or in combination with SIRPα-specific monoclonal antibodies.^[17]^ Induction of macrophage-mediated-ADCP also is a key mode for acute lymphoblastic leukemia treatment.^[18]^ These findings suggest that ADCP is a promising target for cancer immunotherapy.^[19]^ However, the function and mechanisms of the ADCP and its relevant genes remain unclear. Therefore, in the present study, we explored the prognostic value and constructed an ADCP-related gene signature to predict the prognosis of patients with STAD.

We identified 27 ADCP-related key genes that were significantly associated with OS in patients; these genes were enriched in mitochondria-related signaling pathways. The mitochondria are closely associated with ADCP. Li et al reported that mitochondrial fission enhances the ADCP effect of macrophages, thereby playing an important role in the phagocytosis of cancer cells.^[20]^ During ADCP, the cell membrane of macrophages is in an unstable state, and the DNA sensor AIM2 can capture mitochondrial DNA from cancer cells through gaps in the membranes of macrophages.^[8]^ In addition, we screened 8 genes that were significantly associated with prognosis using Cox LASSO regression. Among these, ARID2 and CAPRIN1 are involved in transcriptional regulation,^[21,22]^ AK2 and CAB39 primarily participate in cellular metabolism,^[23,24]^ and AXL, BCL6, and C5AR1 mainly function in immune responses.^[25–27]^ The experimental analysis demonstrated that high expression of C5AR1 in tumors compared to adjacent-normal tissues in STAD. These biological functions play key roles in the ADCP. We then constructed a risk assessment model and classified patients into high- and low-risk groups based on the optimal threshold. This model showed excellent ability in predicting prognosis in the training and validation sets.

Moreover, we showed that patients in the different risk groups had distinct molecular and immune microenvironmental features that may explain their different prognoses. We observed higher immune score and lower stromal score in the high-risk group compared to low-risk group. Furthermore, we observed significant decreased the numbers of CD8 + T cells, monocytes, and neutrophils, but increased the numbers of plasma cells in high-risk group compared to low-risk group. CD8 + T cells in tumors have been extensively investigated; higher CD8 + T cell levels in tumors are correlated with improved survival in patients with STAD.^[28,29]^ Moreover, Nechita et al reported that lower neutrophil levels in tumors were associated with more aggressive tumors.^[30]^

Several therapeutic antibodies exert their effects through the ADCP. Furthermore, a significant low in the RNA expression levels of CD274, CD47, HLA-A, HLA-DOA, HLA-DOB, HLD-DRA, HLA-DRB1, HLA-F, HLA-G, ICOS, SIRPA, and TNFRSF4 in high-risk than low-risk group has been observed. Disrupting CD47 to SIRPα is a promising immunotherapeutic strategy for advanced cancers by enhancing ADCP.^[31]^ Therapeutic antibodies can bind to both cancer cells and macrophages, inducing phagocytosis of cancer cells. Tobias Zeller et al have revealed that checkpoint blockade of CD47 can significantly enhance ADCP in macrophages by blocking the “Don’t Eat Me!” signals.^[32]^ Zhai et al also found that the PD-1 immune checkpoint leads to the elimination of PD-1-positive cancer cells through ADCP and complement-dependent cytotoxicity mechanisms.^[33]^ Given the pivotal role of ADCP in tumor immunotherapy, we further investigated the predictive performance of the ADCP-related signature for response to ICIs. We found that immune-related genes exhibited different expression patterns between the 2 groups. Moreover, some widely accepted predictors of response to immunotherapy showed higher scores in the low-risk group. These results suggest that our ADCP-related signature may predict the response of patients with STAD to immunotherapy.

This study has several limitations. It was conducted in silico and requires further external validation of the role of ADCP-related signatures in ICI treatment. Additionally, factors such as TMB and TIDE can only serve as predictors and cannot fully substitute for the actual patient response to immunotherapy.^[34,35]^ Further in vitro and in vivo studies are essential to elucidate the biological mechanisms of ADCP in STAD. Despite these limitations, our study provides robust evidence supporting the association of ADCP with prognosis in STAD, particularly for predicting response to immunotherapy.

## 5. Conclusion

In the present study, using bioinformatics analysis of multiple genomic datasets, we constructed an ADCP model consisting of 8 genes associated with the prognosis and response to immunotherapy for STAD and provided insights into the biological mechanisms underlying this predictive function.

## Author contributions

**Conceptualization:** Zhuguang He, Zhiming Chen, Xinyue Tang, Ang Wang.

**Data curation:** Xiaochuan Li, Hongjian Wang, Xiaofeng Li, Zhiming Chen, Xinyue Tang, Ang Wang.

**Formal analysis:** Xiaochuan Li, Hongjian Wang, Xiaofeng Li, Zhiming Chen, Xinyue Tang, Ang Wang.

**Investigation:** Xiaochuan Li, Hongjian Wang, Xiaofeng Li.

**Methodology:** Xiaochuan Li, Hongjian Wang, Xiaofeng Li.

**Software:** Xiaochuan Li, Hongjian Wang, Xiaofeng Li.

**Supervision:** Miaoen Zeng, Zhuguang He, Linjie Song.

**Validation:** Miaoen Zeng, Zhuguang He, Linjie Song.

**Writing – original draft:** Xiaochuan Li, Hongjian Wang, Xiaofeng Li, Zhiming Chen, Xinyue Tang, Ang Wang.

**Writing – review & editing:** Xiaochuan Li, Hongjian Wang, Xiaofeng Li, Zhiming Chen, Xinyue Tang, Ang Wang.

## Supplementary Material


